# Structural and Functional Abnormalities of Default Mode Network in Minimal Hepatic Encephalopathy: A Study Combining DTI and fMRI

**DOI:** 10.1371/journal.pone.0041376

**Published:** 2012-07-20

**Authors:** Rongfeng Qi, Qiang Xu, Long Jiang Zhang, Jianhui Zhong, Gang Zheng, Shengyong Wu, Zhiqiang Zhang, Wei Liao, Yuan Zhong, Ling Ni, Qing Jiao, Zongjun Zhang, Yijun Liu, Guangming Lu

**Affiliations:** 1 Department of Medical Imaging, Jinling Hospital, Clinical School of Medical College, Nanjing University, Nanjing, Jiangsu Province, China; 2 Key Laboratory for NeuroInformation of Ministry of Education, School of Life Science and Technology, University of Electronic Science and Technology of China, Chengdu, Sichuan Province, China; 3 Department of Biomedical Engineering, Zhejiang University, Hangzhou, Zhejiang Province, China; 4 Medical Imaging Institute of Tianjin, Tianjin, China; 5 Center for Cognition and Brain Disorders and the Affiliated Hospital, Hangzhou Normal University, Hangzhou, Zhejiang Province, China; 6 Department of Psychiatry, University of Florida McKnight Brain Institute, Gainesville, Florida, United States of America; University of Manchester, United Kingdom

## Abstract

**Background and Purpose:**

Live failure can cause brain edema and aberrant brain function in cirrhotic patients. In particular, decreased functional connectivity within the brain default-mode network (DMN) has been recently reported in overt hepatic encephalopathy (HE) patients. However, so far, little is known about the connectivity among the DMN in the minimal HE (MHE), the mildest form of HE. Here, we combined diffusion tensor imaging (DTI) and resting-state functional MRI (rs-fMRI) to test our hypothesis that both structural and functional connectivity within the DMN were disturbed in MHE.

**Materials and Methods:**

Twenty MHE patients and 20 healthy controls participated in the study. We explored the changes of structural (path length, tracts count, fractional anisotropy [FA] and mean diffusivity [MD] derived from DTI tractography) and functional (temporal correlation coefficient derived from rs-fMRI) connectivity of the DMN in MHE patients. Pearson correlation analysis was performed between the structural/functional indices and venous blood ammonia levels/neuropsychological tests scores of patients. All thresholds were set at *P*<0.05, Bonferroni corrected.

**Results:**

Compared to the healthy controls, MHE patients showed both decreased FA and increased MD in the tract connecting the posterior cingulate cortex/precuneus (PCC/PCUN) to left parahippocampal gyrus (PHG), and decreased functional connectivity between the PCC/PCUN and left PHG, and medial prefrontal cortex (MPFC). MD values of the tract connecting PCC/PCUN to the left PHG positively correlated to the ammonia levels, the temporal correlation coefficients between the PCC/PCUN and the MPFC showed positive correlation to the digital symbol tests scores of patients.

**Conclusion:**

MHE patients have both disturbed structural and functional connectivity within the DMN. The decreased functional connectivity was also detected between some regions without abnormal structural connectivity, suggesting that the former may be more sensitive in detecting the early abnormalities of MHE. This study extends our understanding of the pathophysiology of MHE.

## Introduction

Minimal hepatic encephalopathy (MHE) refers to a transitional stage between non-hepatic encephalopathy cirrhotic patients and overt HE (OHE), which is used to classify a subpopulation of cirrhotic patients with no obvious clinical manifestation of HE but can be identified with neuropsychological examination [1], [2], [3]. MHE is considered to be associated with poor quality of life and increased work disability, and has some propensity to develop into OHE [3]. HE is reversible with appropriate treatment in the initial phase, e.g., treatment with lactulose [4] or rifaximin [5] may improve cognitive function and health-related quality of life for MHE patients. However, how MHE leads to brain abnormality remains unclear, the investigation of MHE therefore has great potential to improve the understanding of disease pathophysiology and aid accurate diagnosis.

Imaging plays an important role in detecting structural and functional abnormality of the brain in HE patients. Findings of diffusion tensor imaging (DTI) [6], [7], [8] have established low grade cerebral edema in cirrhosis that is otherwise difficult to detect with conventional MR imaging, which may be resolved after liver transplantation [8] or successful medical treatment [6]. As a recent focused theme of study in contemporary cognitive and clinical neuroscience, the brain default-mode network (DMN) during “rest” is thought to be engaged in the maintenance of the baseline brain activities related to cognitions of self-awareness, episodic memory and interactive modulation between the internal mind activities and external tasks [9], [10], [11], [12]. The DMN comprises the posterior cingulate cortex/precuneus (PCC/PCUN), medial prefrontal cortex (MPFC), bilateral inferior parietal lobule (IPL), inferior temporal cortex (ITC), and parahippocampal gyrus (PHG). Investigating the effects of disease on the DMN may be particularly important for understanding the impact of disease on the brain [13], which have been used in various mental disorders [14], even in the early stage of these diseases, such as Alzheimer's disease [15] and HE [16]. A recent resting-state functional MRI (rs-fMRI) study by Zhang et al. reported decreased functional connectivity within the DMN in OHE patients [17].

In this study, we hypothesize that both structural and functional connectivity within the DMN is disturbed in MHE, the early stage of HE. To test our hypothesis, we combined DTI tractography with rs-fMRI to investigate the structural and functional connectivity within the DMN in MHE patients. DTI fiber tractography is a direct way to depict the structural connectivity of brain network [18], [19]. This approach can be used to estimate the routes taken by fiber pathways connecting different brain regions of the human brain. Functional connectivity is based on the temporal coherence of spontaneous blood oxygenation level-dependent (BOLD) fluctuations within brain network that are either anatomically connected or not [20]. It is widely accepted that the functional connectivity reflects the direct or indirect (i.e. via a third region) structural connectivity architecture [20], [21], [22], enriching our understanding of human brain networks. Therefore, studying the changes in functional and structural connectivity within the DMN may improve our understanding of the neural underpinnings of the MHE via multi-modality MRI.

## Materials and Methods

### Subjects

This study was approved by the Medical Research Ethics Committee of Jinling Hospital and Clinical School of Medical College at Nanjing University. Written informed consents were obtained from all the participants before the study. Twenty hospitalized MHE patients (13 male, 7 women, mean age: 55.10±7.22 years) were included in this study. The inclusion criteria for recruitment of the patients were as follows: the patients with clinical proven hepatic cirrhosis, without clinical manifestation of HE, had abnormal neuropsychological tests scores, who could finish the MR exam without any MRI contraindication, age 18 years or older. Exclusion criteria for the subjects included any drug abuse history, any brain lesions such as tumor, stroke assessed on basis of medical history and conventional MRI, or translation more than 1.0 mm or rotation than 1.0° during MR scanning.

The diagnosis of MHE was made according to the recommendation by the working party of 11^th^ world congress of gastroenterology [3]. All patients underwent two neuropsychological tests: number connecting-A (NCT-A) and digit symbol test (DST) [23], [24]. NCT-A tests for psychomotor speed, and worse performance is indicated by a longer time for completion. DST tests for psychomotor speed, attention, and visual memory. The number of correctly transcribed symbols indicates performance, i.e., a low score means poor performance. When the scores of at least one test were beyond 2SD (standard deviation) of mean value of age-matched healthy controls, the cirrhotic patients could be regarding having MHE [24], [25]. Laboratory parameters including prothrombin time, protein metabolism tests, venous blood ammonia were obtained from all patients to assess the severity of liver disease, within one week before MR scanning. The grade of hepatic function was determined according to the Child-Pugh score [26], [27]. Of these 20 MHE patients, 9 patients had Child-Pugh grade A and 11 patients had Child-Pugh grade B. All patients were right-handed. The etiology of cirrhosis was hepatitis B in 18 patients, hepatitis A and C in one patient each. Twenty age-and gender-matched right-handed healthy controls from local community were recruited in this study (13 male, 7 women, and mean age: 53.95±8.09 years). All healthy controls had no diseases of the liver and other systems, with no abnormal findings in abdominal ultrasound scans (performed within one week before MR scan) and conventional brain MR imaging. The healthy controls were interviewed by the psychiatrist in our hospital to confirm that they all had no any current and history of psychiatric illness (such as depression, social anxiety disorder) and substance intake; furthermore, their first-degree relatives also had no any history of psychiatric illness. All controls underwent neuropsychological tests (NCT-A, and DST) before the MR scanning. No laboratory tests were performed thus unavailable for them. Demographics and clinical data for all the 40 participants were summarized in Table 1.

**Table 1:** Demographics and clinical data of MHE patients and healthy controls.

Protocols	HC (n = 20)	MHE (n = 20)	*P* value
Sex (M/F)	13/7	13/7	1^a^
Age (±SD), y	55.10±7.22	56.57±9.19	0.64^b^
Venous blood ammonia (in umol/L)		69.06±26.13	
Child-Pugh scale			
A		9	
B		11	
C		0	
NCT-A (s)	46.68±7.39	72.80±16.71	<0.001^b^
DST	42.29±8.91	23.15±8.17	<0.001^b^

Values are expressed as mean ± SD. HC = healthy control; MHE = minimal hepatic encephalopathy. NCT-A = number connecting-A; DST = digit symbol test.

a The *P* value for gender distribution in the two groups was obtained by chi-square test.

b The *P* value for age and neuropsychological tests difference between the two patients groups was obtained by two sample *t* test.

### MRI data acquisition

MRI data were acquired on a 3 Tesla MR scanner (TIM Trio, Siemens Medical Solutions, Erlangen, Germany). The participants were instructed to lie quietly and keep their eyes closed but be awake in the MR scanner. Foam pad was used to minimize the head motion of all subjects. First, high-resolution T1-weighted 3D anatomical images were obtained in the sagittal orientation using a magnetization-prepared rapid gradient-echo sequence (TR/TE = 2300 ms/2.98 ms, flip angle = 9°, 191 slices, FOV = 256×256 mm^2^, acquisition matrix = 256×256, slice thickness = 1 mm). Functional images were subsequently obtained aligned along the anterior commissure-posterior commissure line with a single-shot, gradient-recalled echo planar imaging sequence (TR/TE = 2000 ms/30 ms, FOV = 240×240 mm^2^, flip angle = 90°, matrix = 64×64, voxel size = 3.75×3.75×4 mm^3^). A total of 250 brain volumes were collected, resulting in a total scan time of 500 s. Then, the diffusion tensor images were obtained using a spin echo-based echo planar imaging sequence in contiguous axial planes, including 20 volumes with diffusion gradients applied along 20 non-collinear directions (b = 1000 s/mm^2^) and one volume without diffusion weighting (b = 0 s/mm^2^). Each volume consisted of 30 contiguous axial slices covered the whole brain (TR = 4100 ms, TE = 93 ms, flip angle = 90°, FOV = 240×240 mm^2^, matrix size = 128×128, voxel size = 1.8×1.8×4 mm^3^).

### Data preprocessing

For DTI images of each subject, 20 diffusion-weighted images were first registered to B0 image (b = 0 s/mm^2^) using SPM 8 (http://www.fil.ion.ucl.ac.uk/spm) and then corrected for difference in spatial distortion due to eddy currents using FMRIB's Diffusion Toolbox (FDTv2.0) as implemented in FMRIB's Software Library (FSL v4.1; www.fmrib.ox.ac.uk/fsl) [28].

For fMRI images, preprocessing were performed using SPM8 software package. The first 10 images were excluded for magnetization to reach equilibrium. The remaining 240 consecutive volumes were used for data analysis. Slice-timing adjustment and realignment for head-motion correction were performed. No translation or rotation parameters in any given data set exceeded 1.0 mm or 1.0°. We also evaluated the group differences in translation and rotation of head motion according to the following formula [29]: Head Motion/Rotation = 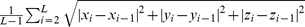 where *L* is the length of the time series (*L* = 240 in this study), *xi*, *yi* and *zi* are translations/rotations at the *i*th time point in the *x*, *y* and *z* directions, respectively.

The results showed that the two groups had no significant differences in image quality (two sample *t* test, *t* = 0.132, *P* = 0.892 for translational motion, and *t* = 0.592, *P* = 0.577 for rotational motion). The functional images were then spatially normalized to standard stereotaxic coordinates of the standard Montreal Neurological Institute (MNI) and resampled into voxel size of 3×3×3 mm^3^, and then smoothed by convolution with an isotropic Gaussian kernel (FWHW = 8 mm).

### DMN extraction

To extract the regions of interest (ROIs) within the DMN in both groups, independent component analysis (ICA) was first applied to decompose the smoothed data of each individual in control and MHE groups into 42 and 39 independent components (ICs) respectively with the Infomax algorithm using the GIFT software (http://icatb.sourceforge.net/, Vision 2.0d). The number of ICs was determined by a dimension estimation using the minimum description length (MDL) criterion [30]. Healthy control and MHE groups in this study were estimated separately to avoid the specific resting-state network pattern from each group to be mixed [31], [32]. To each IC, the time courses correspond to the waveform of a specific pattern of coherent brain activity and the intensity of this pattern of brain activity across the voxels was expressed by the associated spatial map [12]. Then, using the GIFT software, the DMN component of each subject was selected based on the largest spatial correlation with a prior DMN template which included bilateral IPL, ITG, PHG, as well as PCC/PCUN, MPFC. This DMN template was provided by Dr. Liao (Center for Cognition and Brain Disorders and the Affiliated Hospital, Hangzhou Normal University, Hangzhou, China), which has been recruited in previous studies [29], [33]. To display the voxels that contributed most strongly to a particular IC, the intensity values in each spatial map were converted to *z* values. The *z*-values here reflect the degree to which the time courses of a given voxel correlate to the time courses of each special IC [12]. After extracting the DMN from all subjects, a second-level random-effects statistical analysis was performed for the DMN of each group using one-sample *t* test. Significant clusters were thresholded with a false discovery rate (FDR) at *P*<0.01 (corrected for multiple comparisons across the whole brain). Regions of interest (ROIs) for the PCC/PCUN, MPFC, and bilateral IPL, PHG, and ITG were selected from the DMN map of the control group using the xjView toolbox [33] (see Table 2 for details of these ROIs). These eight ROIs were used for the subsequent analyses in both the patient and the control groups.

**Table 2:** Details on the brain regions in the DMN map (P<0.01, FDR corrected) from the healthy controls and MHE patients.

Anatomical region	Hemisphere	MNI coordinates (x, y, z)	Brodmann's area	Cluster size (Voxels)	*t* value
**Healthy controls**					
PCC/PCUN	L/R	0, −51, 30	7, 23, 26, 30	2212	15.32
IPL	L	−68, −66, 30	39, 40	359	16.17
	R	57, −63, 27	39, 40	279	15.15
PHG	L	−27, −24, −15	35, 36	86	6.51
	R	30, −30, −15	35, 36	50	4.77
ITG	L	−57, −21, −18	20, 21,38	522	9.50
	R	54, 0, −33	20, 21,38	488	9.76
MPFC	L/R	3, 42, −18	8, 9, 10, 11, 32	3183	13.64
**MHE patients**					
PCC/PCUN	L/R	6, −51, 33	7, 23, 26, 30	2389	16.09
IPL	L	−45, −72, 42	39, 40	304	15.11
	R	51, −66, 30	39, 40	318	13.94
PHG	L	−27, −33, −15	35, 36	60	5.97
	R	30, −27, −18	35, 36	55	6.67
ITG	L	−60, −9, −18	20, 21,38	424	8.91
	R	60, −6, −24	20, 21,38	356	9.15
MPFC	L/R	−6, 48, −9	8, 9, 10, 11, 32	3141	10.46

DMN = default mode network; FDR = false discovery rate; MHE = minimal hepatic encephalopathy; PCC/PCUN = posterior cingulate cortex/precuneus; MPFC = medial prefrontal cortex; PHG = parahippocampal gyrus; MNI = Montreal Neurological.

### Structural connectivity within the DMN

#### Fiber tracking

Whole-brain fiber tracking was performed in the DTI native space for each subject using the Diffusion Toolkit and TrackVis software (http://www.trackvis.org/), with an interpolated streamline propagation algorithm [33], [34]. Path tracing proceeded until either the FA fell below 0.15, or the minimum angle between the current and the previous path segment was higher than 35° [33], [35].

#### Fiber extraction within DMN

As the eight ROIs within DMN were derived from the normalized MNI space, inverse transformation (*T* ^−1^) was applied to the eight ROIs in the normalized MNI, resulting in the subject-specific ROIs in the native space of DTI [20], [33]. Fiber bundles connecting each pair of ROIs were then extracted from the total collection of brain fibers. This was done as follows: first, an initial ROI was selected, and the tracts that reached the first ROI were chosen from all fibers; second, another ROI was retrieved from the rest ROIs. Only those tracts that reached the second ROI were picked from the resulting tracts of the previous step; finally, fibers that were anatomically implausible were identified visually and removed. The remaining fiber bundles connecting each pair of ROIs were prepared for the subsequent analyses. In the present study, four basic indices of fiber connectivity obtained from TrackVis [33], [36] were performed into the structural connectivity analysis to determine whether there were any abnormalities in patients when compared to the healthy controls, including path length, tract count, mean fractional anisotropy (FA) and mean diffusivity (MD) of each fiber pathways within DMN.

#### Functional connectivity within the DMN

To acquire the temporal correlation coefficients between each pair of ROIs within the DMN in the DTI native space, first, the functional ROIs were also inverse transformed to the native space in the same way as DTI on the aforementioned eight ROIs within DMN; second, to extract the time series for cerebrospinal fluid (CSF) and white matter (WM), individual's T1 weighted anatomical images were segmented using SPM8, with the threshold of the segmented probability images setting at 80% [33]. Then the segmented CSF and WM were coregistered to the individual's B0 images (*b* = 0 s/mm^2^), to create subject-specific CSF and WM templates; third, the time series were extracted from each ROI in the DTI native space, and then removed several sources of spurious variance by linear regression, including six head motion parameters, and average signals from the subject-specific CSF, WM, and whole brain masks. By doing this regression, fluctuations unlikely to be involved in specific regional correlation were removed; fourth, the residuals time series were band filtered (0.01–0.08 Hz), and the functional connectivity of brain regions within the DMN was computed by calculating the temporal correlation coefficients between the residuals time series in each pair of regions.

### Statistical analysis

Structural and functional connectivity for each pair of ROIs was compared between patient and control groups. Specifically, structural connectivity (including path length, tract count, fractional anisotropy, mean diffusivity), and functional connectivity (temporal correlation coefficient [after Fisher-*z* translation]) were investigated by two sample *t*-tests (*P*<0.05, corrected for multiple comparison using the Bonferroni correction for the number of tracts that showed both structural and functional connectivity in all subjects, three tracts [fiber tracks which connecting the PCC/PCUN with MPFC, and bilateral PHG] were considered in the present study).

To investigate the potential effect of venous blood ammonia on structural and functional connectivity within DMN in MHE patients, and the relationship between their neuropsychological tests scores and DMN connectivity indices, structural/functional indices that showed difference between patients and controls were correlated against the venous blood ammonia levels, and the scores of NCT-A and DST, using the Pearson correlation analysis. Correlation analysis was performed using SPSS 16.0 (SPSS Inc., Chicago, IL), the threshold was set at a significance level of *P*<0.05, corrected for multiple comparison using the Bonferroni correction for the number of tracts that showed either different structural or functional connectivity between patients and controls (two tracts were considered in the Bonferroni correction in this study [fiber tracks which connecting the PCC/PCUN with MPFC, and left PHG]) [33].

## Results

### Spatial pattern of DMN in each group

**Figure 1:**
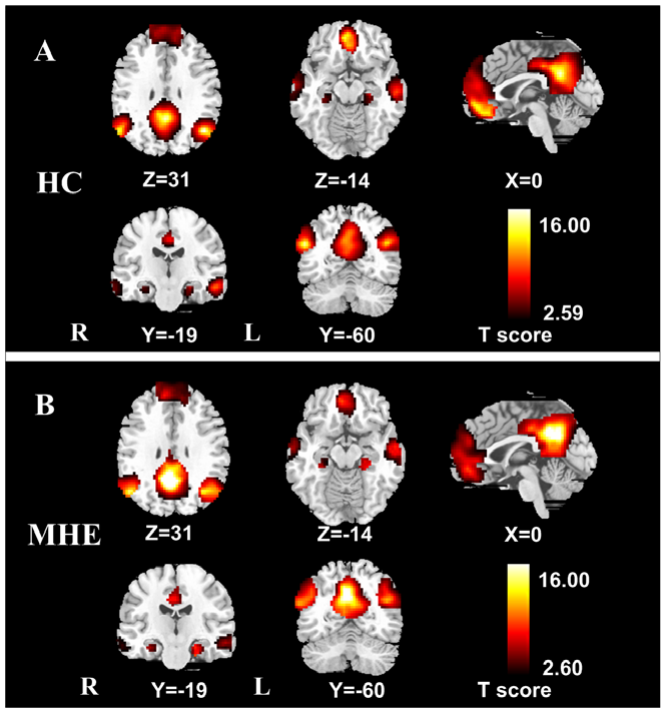
Within-group DMN maps of healthy controls and MHE patients. A: DMN in the healthy controls (*t*>2.59, *P*<0.01, FDR corrected). B: DMN in the MHE patients (*t*>2.60, *P*<0.01, FDR corrected). The DMN in healthy controls and MHE patients shows functional connectivity among the PCC/PCUN, the MPFC, the bilateral IPL, PHG, and ITG. DMN = default mode network; MHE = minimal hepatic encephalopathy; FDR = false discovery rate. PCC/PCUN = posterior cingulate cortex/precuneus; MPFC = medial prefrontal cortex; IPL = inferior parietal lobule; PHG = parahippocampal gyrus; ITC = inferior temporal cortex.

The random-effect analysis of the single-subject DMN maps revealed a typical spatial pattern of this DMN in both groups (*P*<0.01, FDR corrected) (Figure 1). The DMN pattern in the controls (Figure 1A, Table 2) showed functional connectivity among the PCC/PCUN, the MPFC, the bilateral IPL, PHG, and ITG. The DMN pattern of the patients (Figure 1B, Table 2) largely included the same brain regions as the controls.

### Structural connectivity within DMN

**Figure 2:**
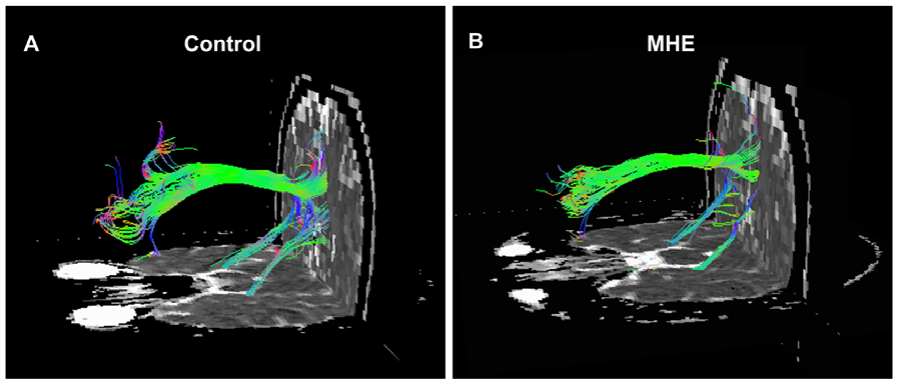
Example of DTI fiber tractography on one healthy control and one MHE patient. Only three fiber bundles connecting the PCC/PCUN and MPFC, bilateral PHGs were detected in all the subjects. The color-coding of the obtained fibers is based on the standard RGB (Red, Green, Blue) code applied to the vector at every segment of each fiber. Red indicates the medio-lateral plane. Green indicates the dorsoventral orientation. Blue indicates the rostro-caudal direction. DTI = diffusion tensor imaging; MHE = minimal hepatic encephalopathy; PCC/PCUN = posterior cingulate cortex/precuneus; MPFC = medial prefrontal cortex; PHG = parahippocampal gyrus.

Figure 2 showed two examples of fiber bundles connecting the PCC/PCUN to the MPFC, and to the bilateral PHG in a healthy control and a MHE patient, respectively. Three fiber pathways were detected in all the healthy controls and patients: the cingulum tracts connecting the PCC/PCUN to MPFC, and fiber tracts connecting the PCC/PCUN to left and right PHG respectively. The left and right superior frontal-occipital fasciculus connecting the left and right IPL to the MPFC, respectively, were detected in 1/3 (left/right) out of 20 healthy controls and in 2/3 (left/right) out of 20 MHE patients. The tracks that connected the PCC/PCUN to the left and right ITG, respectively, were detected in 3/2 (left/right) of 20 controls and in 3/2 (left/right) of 20 MHE patients. Thus, we only further compared the structural indices of three tracts detected in all subjects (the fiber bundles located between the PCC/PCUN and MPFC, bilateral PHG) in the same way as performed in previous studies [33]. Values were given as mean ± standard deviation (SD). The results of two-sample *t* test showed both decreased mean FA (mean ± SD: 0.240±0.026 and 0.279±0.030 for the patients and controls respectively; *t* = 3.421, *P* = 0.002) (Figure 4C) and increased MD [mean ± SD: (1.258±0.152)×10^−3^ mm^2^/s and (1.140±0.109)×10^−3^ mm^2^/s for the patients and controls respectively; *t* = 2.839, *P* = 0.007] (Figure 4D) on the fiber bundle connecting the PCC/PCUN to left PHG in MHE patients when compared with controls. Path length or tract count of this fiber demonstrated no significant difference (*P*>0.05) (Figure 4A, 4B). In addition, no difference of any structural connectivity indices were found in other two fibers bundles (all *P*>0.05).

### Functional connectivity within DMN

**Figure 3:**
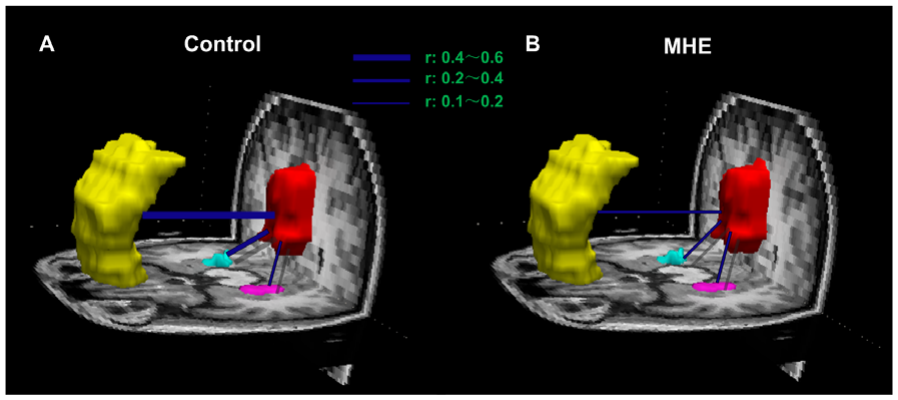
Functional connectivity within the DMN in healthy control and MHE patient groups. Averaged temporal correlation coefficient *r* (Blue) across all subjects in the healthy control group (A) and in the MHE patient group (B). Compared with healthy controls, MHE patients show decreased temporal correlation coefficients between the PCC/PCUN and the MPFC, as well as between the PCC/PCUN and the left PHG. MPFC is color-coded yellow, PCC/PCUN color-coded red, the left PHG color-coded pink, the right PHG color-coded cyan. DMN = default mode network; MHE = minimal hepatic encephalopathy. PCC/PCUN = posterior cingulate cortex/precuneus; MPFC = medial prefrontal cortex; PHG = parahippocampal gyrus.

**Figure 4:**
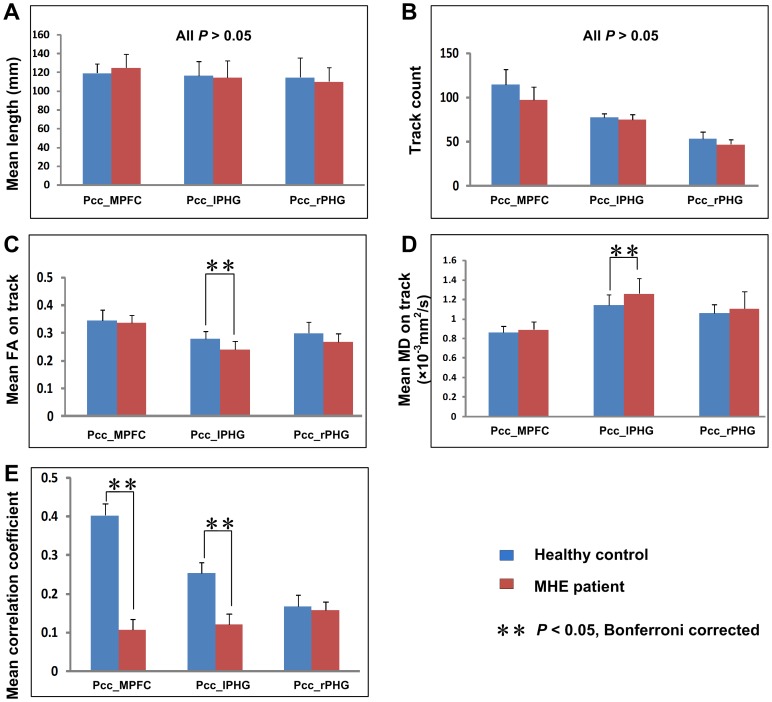
Comparison of structural and functional connectivity between MHE patients and healthy controls. Compared to the healthy controls, MHE patients show both decreased FA and increased MD in the tract connecting the PCC/PCUN to left PHG, and decreased functional connectivity between the PCC/PCUN and left PHG, and MPFC. For each connection, differences are setting at the significant level of corrected *P*<0.05 (marked with **) with Bonferroni correction. Error bars represent the standard deviation of the measurements. MHE = minimal hepatic encephalopathy; PCC/PCUN = posterior cingulate cortex/precuneus; PHG = parahippocampal gyrus; MPFC = medial prefrontal cortex; FA = fractional anisotropy; MD = mean diffusivity.

Figure 3 showed averaged temporal correlation coefficient *r* in control (Figure 3A) and patient (Figure 3B) groups, respectively. Based on the results from above-mentioned structural connectivity, we only examined three pairwise functional connectivity between the PCC/PCUN and the MPFC, bilateral PHG, which showed inter-regional structural connectivity in all subjects. The comparison results showed that temporal correlation coefficients between the PCC/PCUN and the MPFC (mean ± SD: 0.108±0.027 and 0.403±0.030 for the patients and controls respectively; *t* = 4.739, *P*<0.001), as well as between the PCC/PCUN and the left PHG (mean ± SD: 0.121±0.027 and 0.253±0.028 for the patients and controls respectively; *t* = 2.863, *P* = 0.007) were decreased significantly in MHE patients when comparing to healthy controls (Figure 4E).

### Pearson correlation results

**Figure 5:**
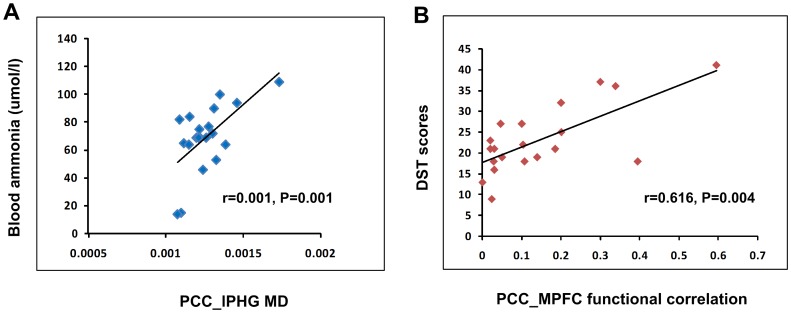
Correlation between the structural/functional indices and clinical markers of MHE patients. Pearson correlation results reveal that in MHE patients, the MD values of the tracts connecting PCC/PCUN to the left PHG positively correlate to the blood ammonia levels, the temporal correlation coefficients between PCC/PCUN and the MPFC show positive correlation to the DST scores (*P*<0.05, Bonferroni corrected). MHE = minimal hepatic encephalopathy; MD = mean diffusivity; PCC/PCUN = posterior cingulate cortex/precuneus; PHG = parahippocampal gyrus; MPFC = medial prefrontal cortex; DST = digital symbol test.

Pearson Correlation results (Figure 5) revealed that in MHE patients, the MD of the fiber bundle connecting PCC/PCUN to the left PHG positively correlated to the blood ammonia levels (*r* = 0.616, *P* = 0.004) (Figure 5A), the temporal correlation coefficients between PCC/PCUN and the MPFC showed positive correlation to the DST scores (*r* = 0.705, *P* = 0.001) (Figure 5B). Other structural and functional indices displayed no significant correlation with blood ammonia levels or neuropsychological performance.

## Discussion

In the present study, by combining the structural and functional connectivity to investigate the abnormalities of DMN in MHE patients, we found that MHE patients have both disturbed structural and functional connectivity within the DMN, and the latter may be more sensitive in detecting the brain abnormality of MHE. In addition, we also found that some structural and functional connectivity indices of MHE patients had significant correlation with the ammonia levels and neuropsychological tests scores. This study linking connectivity at rest with brain structure and function extends our understanding of the neuropathophysiology of MHE.

### Structural and functional connectivity within DMN

Neuronal activity is transmitted via neuronal fibers. White matter tracts are bundles of huge numbers of axons that connect large neuronal population over long distances [37]. Although the exact relationship between functional connectivity from resting-state MRI and structural connectivity from DTI tractography has not been explored in detail within DMN, some recent studies have indicated that the structural connectivity is the material substrate of functional connectivity [20], [38], [39]. Kaiser et al. also demonstrated that a long-distance connectivity may be important for complex neural systems [40]. DMN, which is thought to reflect the brain intrinsic activities, is critical to brain function [41]. Combining the DTI tractography and functional connectivity MRI to investigate the DMN of brain diseases such as the mesial temporal lobe epilepsy [33] may greatly enhance the understanding of the neuro-pathophysiological mechanism [20]. However, to our knowledge, no such studies linking structural and functional connectivity have been performed in MHE patients.

### Abnormal structural connectivity within DMN in MHE

Low-grade cerebral edema is considered to be responsible for the neuropsychological abnormalities in cirrhotic patients, primarily resulting in increased MD in the widespread cortical gray and white matter, as well as decreased FA mainly occurred in the bilateral frontal and occipital white matter [6], [7]. To investigate this brain edema caused by chronic liver failure, previous DTI studies often used the hypotheses-based region of interest (ROI) analysis method, or a three-dimensional voxel-based analysis method [7]. To the best of our knowledge, no studies have revealed the changes of certain white fiber bundles which are the structural bases of information processing and transporting among linked brain regions in MHE patients, such as the fiber pathways between the DMN regions.

The current study mainly focused on the cingulum and the tracts connecting the PCC/PCUN to bilateral PHG interconnecting the brain regions within the DMN. We found that the tract connecting the PCC/PCUN to left PHG showed both decreased FA and increased MD in MHE patients compared with healthy controls, but without significantly changed path length, tracts count. These findings further supported the theory of the role of the low cerebral edema of extracellular origin rather than microstructural damage in chronic liver faliure [6], [7]. According, the decreased FA and increased MD of the fiber connecting PCC/PCUN to PHG in MHE patients in this study may be explained by the less dense packing of axonal fibers due to the cerebral edema. In this study, the fibers connecting PCC/PCUN to right PHG and MPFC showed slightly higher MD and lower FA in MHE than those in controls but without significant difference among both groups (all *P*>0.05). The consistency of these asymmetric changes of FA and MD between hemispheres in MHE in the present study needs further investigation in a larger sample size. Increased MD and decreased FA have been reported in many previous ROI-based DTI studies of chronic cirrhotic patients [6], [8], [42], in which the abnormalities may revert to normal following effective therapy [43] or liver transplantation [8]. So the findings of brain fiber bundles abnormalities but without significant microstructural damages in MHE patients extend our understanding of the pathophysiology of this disease.

### Disturbed functional connectivity within DMN in MHE

Functional connectivity is typically interpreted as the temporal synchronization of low-frequency fluctuations arising from spontaneous neuronal activities in distant brain regions [10]. In the present study, the functional connectivity between the PCC/PCUN and MPFC, and left PHG decreased significantly in MHE patients. The disturbed functional connectivity within DMN of HE patients has been reported in two previous studies [16], [44]. Zhang et al. first reported a decreased functional connectivity of MPFC, PCC/PCUN and IPL within the DMN in overt HE patients in a 1.5 T resting-state fMRI study [17]. The second study by Chen et al. demonstrated that in a group of cirrhotic patients, the decreased functional connectivity of PCC to MPFC, left IPL, and left middle temporal gyrus still persisted after clinical recovery from previous episodes of overt HE [44]. As the effect of HE is global, whether there is an asymmetry of left and right hemispheric functional connectivity as suggested in the present and a previous fMRI studies [44] needs further studies in the future. So our findings of abnormal functional connectivity within DMN in MHE patients fit well with previous studies, which suggested that the DMN abnormality was already present in the early phase of the HE.

In addition, taking into account that PCC/PCUN and MPFC in MHE patients in the present study showed decreased functional connectivity but no significantly changed inter-regional structural connectivity, we speculated that functional connectivity may be more sensitive than structural connectivity in detecting the brain abnormality within DMN in MHE patients. In general, the functional connectivity which measures the blood oxygenation level-dependant fluctuations is thought to be more flexible; while the structural connectivity is relatively stable [35], [45]. The inconsistent changes of structural and functional connectivity are also reported in previous MRI studies, e.g., Zhang et al. reported less affected structural connectivity but widespread impaired functional connectivity in idiopathic generalized epilepsy patients [35]. The findings here need to be confirmed in further studies with more patients.

### Relationship between structural and functional connectivity indices and clinical markers of MHE

In the present study, the positive correlation between MD values of tract connecting PCC/PCUN to left PHG and the blood ammonia levels in MHE patients was partially in accordance with published DTI studies in chronic cirrhotic patients with or without HE [46], [47], suggesting the blood venous ammonia plays an important role in the pathogenesis of HE. We also found a positive correlation between functional connectivity between PCC/PCUN and MPFC and the DST scores. PCC/PCUN is a core hub showed strong correlation with each other brain regions in the DMN, which plays a pivotal role for the DMN [41]. MPFC plays an important role in cognitive control [48]. DST, one of the neuropsychological tests which were most frequently used in defining MHE, mainly tests the domains of attention, psychomotor speed, and visual memory [25]. Disturbance in these domains is common in MHE patients. Chen et al. have demonstrated that the DST scores in MHE patients positively correlated with the regional brain activity in the cuneus and adjacent precuneus [16]. So, these correlation results in the present study extend our understanding of the impact of ammonia on brain connectivity, as well as the correlation between the neuropsychological tests and brain connectivity.

### Study limitations

The current study has some limitations. First, the selection of the seed nodes in the network and the seed size is still an issue for debate in functional connectivity studies. For example, possible differences between controls and patients in network topology should be considered. In the present study we selected the ROI based on the DMN map from the controls, taking into account that reduced connectivity in patients may potentially result in a worse estimation of the ROI locations [33]. Second, in this study, the structural and subsequent functional connectivity analyses were restricted to the three pairs of DMN regions that showing fiber connection in all subjects. However, functional connectivity may exist between regions which do not show direct structural connectivity detected by DTI. Third, in the current study, only two neuropsychological tests were used, but these two tests have been recommended to diagnose MHE by the working party of 11^th^ world congress of gastroenterology [3], in the future, we could include broader spectrum of tests to evaluate the cognition function of cirrhotic patients. In addition, neural structural connectivity may possibly exist between some other pairs of DMN regions with more advanced fiber tracking techniques and instruments. Further studies in the future are warranted to confirm or supplement the findings of this study.

### Conclusion

In summary, this study demonstrated both disturbed functional and structural connectivity within the DMN in MHE patients, which also showed correlation with blood ammonia levels and neuropsychological tests. In addition, the decreased functional connectivity was detected between some DMN regions without abnormal inter-regional structural connectivity, suggesting that the functional connectivity may be more sensitive in detecting the brain abnormality of MHE.
